# EIF4A3-induced circBRWD3 promotes tumorigenesis of breast cancer through miR-142-3p_miR-142-5p/RAC1/PAK1 signaling

**DOI:** 10.1186/s12885-022-10200-7

**Published:** 2022-11-28

**Authors:** Xianguo Meng, Wei Li, Ziqi Meng, Yan Li

**Affiliations:** 1 College of Sports Medicines and Rehabilitation, Shandong First Medical University, No. 6699 Qingdao Road, Jinan, 250118 Shandong China; 2 Accounting Institute, Shandong University of Finance and Economics, No. 7366 East Second Ring Road, Jinan, 250220 Shandong China

**Keywords:** Breast cancer, EIF4A3, CircBRWD3, MiR-142-3p, MiR-142-5p, RAC1-PAK1 signaling

## Abstract

CircBRWD3 is a newly discovered circRNA, and its potential function has not been probed. Here, we aimed to molecularly dissect the role of circBRWD3 in the tumorigenesis and progression of breast cancer (BC). qRT-PCR analysis revealed that circBRWD3 expression was dramatically upregulated in BC tissues, a feature that was positively correlated with the poor prognosis of patients with BC. CircBRWD3 knockdown repressed cell proliferation and metastasis, while promoting cell apoptosis in vitro. Consistently, an in vivo circBRWD3 deficiency model exhibited suppressed tumor metastasis and oncogenesis. On the other hand, circBRWD3 overexpression promoted cancer cell activity and tumorigenesis. Further, mechanistic studies elucidated that circBRWD3 sponged both miR-142-3p and miR-142-5p to modulate RAC1 expression, which subsequently activated the RAC1/PAK1 signaling to facilitate the tumorigenesis and progression of BC. Moreover, we discovered that EIF4A3 facilitated circBRWD3 expression by targeting the upstream of BRWD3 pre-mRNA. In conclusion, our study reveals that circBRWD3 facilitates BC tumorigenesis by regulating the circBRWD3/miR-142-3p_miR-142-5p /RAC1/PAK1 axis. In addition, circBRWD3 expression is positively regulated by an RNA-binding protein, EIFA3. Our results provide valuable scientific data for early diagnosis and therapy for breast cancer patients.

**Supplementary Information:**

The online version contains supplementary material available at 10.1186/s12885-022-10200-7.

## Introduction

Breast cancer (BC) ranks as the second cancer-associated death cause in females worldwide [1]. Despite the therapeutic strategies, such as surgical resection, chemotherapy, anti-hormone therapy, targeted therapy, and endocrinotherapy [2–4], the prognosis of patients is still poor because of drug resistance, metastasis, and recurrence [5, 6]. It is, therefore, imperative to identify novel molecular markers for early diagnosis and clinical treatment.

Circular RNAs (circRNAs) are non-coding RNAs (ncRNAs) and are back-spliced from an upstream 3' site to a downstream 5' site with a covalent loop [7]. This covalence renders circRNAs refractory to RNA exonuclease degradation and therefore more stable than linear RNAs [8]. Bioinformatics analyses coupled with high-throughput sequencing have detected the widespread expression of circRNAs in various species [9]. CircRNAs can regulate multiple cellular processes, including cancer metastasis, cell proliferation, apoptosis, and epithelial-mesenchymal transition [10–12]. Emerging evidence reveals that circRNAs exert vital function in the tumorigenesis of diverse malignant tumors. For example, circ_0025202 plays an anti-oncogenic role in HR-positive BC [13], whilecirc_001783 promotes the tumorigenesis of BC through sponging miR-200c-3p [14]. Moreover, circTADA2As can suppress the metastasis and proliferation of BC via regulating the miR-203a-3p/SOCS3 signaling [15]. CircBRWD3 (hsa_circ_0001936), derived from exons 18 to 22 of BRWD3, is a newly discovered circRNA with rarely studies in cancers. To date, only Yan Wang et al. [16] have confirmed the upregulation of CircBRWD3 in lung cancer in Xuanwei (LCXW), but its regulatory mechanism on LCXW is still unknown. Therefore, whether CircBRWD3 plays a regulatory role in BC remains unstudied.

MicroRNAs (miRNAs) belong to non-coding RNAs (ncRNAs) with about 22 nt in length. MiRNAs are potent gene expression regulators through degrading target mRNAs or repressing protein translation [17]. It has been reported that the functions of miRNAs in tumorigenesis are bimodal, functioning as either oncogenes or tumor suppressors [18–20]. Two endogenous competing RNAs (ceRNAs), miR-142-3p and miR-142-5p, have been implicated in the tumorigenesis of BC [21–24]. In addition to circRNAs and miRNAs, ras-related C3 botulinum toxin substrate 1 (RAC1) is commonly found in mammal tissues and cells, functioning as an epithelial differentiation pleiotropic regulator and involved in apoptosis-related pathways through the production of reactive oxygen species [25]. RAC1 has been implicated in multiple cellular processes, such as cell proliferation, tumor metastasis, and epithelial-mesenchymal transition [26]. The same pathways shared by circRNAs, miRNAs, and RAC1 suggest that they might function coordinately to orchestrate tumorigenesis. In addition, RAC1 has been reported to contribute to the tumorigenesis of BC [27–29]. Thus, RAC1 could be the target gene of miRNAs to regulate the tumorigenesis of BC.

Herein, we set out to investigate the function of circBRWD3 and dissect the relevant molecular mechanism in the progression of BC aiming to identify novel diagnostic and treatment targets for patients with BC.

## Materials and methods

### Tissue samples

The tumor and adjacent noncancerous specimens were surgically dissected from 66 patients with BC at Shandong First Medical University. No chemotherapy or radiotherapy had been performed on these patients before surgery. Cancer and noncancerous regions were checked by two pathologists who were not directly involved in this study. The clinical characteristics of the BC patients were presented in Table 1. All enrolled patients were informed of the aim of this study. The experimental procedures were authorized by the Shandong First Medical University Institutional Ethics Committee. The experiments were performed following the Declaration of Helsinki [30].

**Table 1:** The clinical characteristics of the studied BC patients

Variable	Number	**circBRWD3** expression		*P* value
		Low (33)	High (33)	
Age				
< 40	32	17	15	0.622
≥ 40	34	16	18	
Tumor size (cm)				
< 2	36	25	11	0.012*
≥ 2	30	8	22	
Lymph node metastasis				
No	32	20	12	0.049*
Yes	34	13	21	
TNM stage				
I + II	28	18	10	0.046*
III + VI	38	15	23	
Distant metastasis				
No	30	20	10	0.013*
Yes	36	13	23	
BMI				
18.5–24	32	18	14	0.325
< 18.5	34	15	19	
Smoking				
No	40	19	21	0.614
Yes	26	14	12	

### Cell culture and transfection

A human normal breast epithelial cell (MCF-10A) and four BC cell lines (BT-549, MDA-MB-231, SUM-159, and MDA-MB-453) were obtained from ScienCell Company (San Diego, CA, USA) and were cultured in an RPMI‑1640 medium (Hyclone, USA) with 20% fetal bovine serum (FBS, Hyclone, USA) at 37℃ with 5% CO_2_. The sh-NC, sh-circBRWD3, sh-EIF4A3, OE-vector, OE-circBRWD3, OE-EIF4A3, OE-RAC1, inhibitor control, miR-142-3p inhibitor, miR-142-5p inhibitor, mimics control, miR-142-3p mimics, and miR-142-5p mimics were synthesized by Genechem Company (Shanghai, China) and were transfected into cells using Lipofectamine 3000 reagent (Invitrogen, USA).

### RNase R Treatment assay

Cytoplasmic and nuclear RNAs were isolated by a cytoplasmic and nuclear RNA purification kit (Thermo Fisher Scientific, USA). Total RNAs from BC tissues and cell lines were extracted by the TRIzol reagent (Invitrogen, USA). Then the total RNAs were treated with RNase R (3 U/mg) for 15 min at 37℃. The cDNAs were reverse-transcribed from 2 μg RNA for qRT-PCR.

### Quantitative real-time PCR

The tissue or cell RNAs were isolated by the Trizol reagent (Thermo Fisher Scientific, USA). The genome DNA was removed by RQ DNAase (Promega, Madison, USA) digestion at 37℃ for 30 min followed by extraction by phenol/chloroform/isoamyl alcohol (125:24:1, pH 4.7, sigma). PCR was run with the SYBR Green PCR master mix (Thermo Fisher Scientific, USA) on a Thermal Cycler Dice Real-Time System (Takara, Japan). The gene expression was calculated according to the 2^−ΔΔCt^ method. The primers were listed in Table 2. Each experiment was done in triplicate.

**Table 2:** The mRNA primers for qRT-PCR analyses

Gene	Forward 5′–3′	Reverse 5′–3′
EIF4A3	5′-GCAGTCACGAAGAACAGGGAC-3′	5′-GAGTCGTGGCCTTATCGTTCT-3′
CircBRWD3	5′-CGAGGGACAGCAGTCAGAACA-3′	5′-GTGGCGGAGTCTTCCCTTATT-3′
MiR-142-3p	5′- AGACAGATAGCCCGCAGAGG -3′	5′-GATCTGCTGCCCTTGTGCTGTC-3′
MiR-142-5p	5′- AGCCCGCAGGAGACAGATAG -3′	5′-GCTGCCGATCTTGCTCCTTGGT-3′
U6	5′-CGCTTCCAGCACATATAC -3′	5′-CGCTTCGGCAGCACATATAC -3′
RAC1	5′- TGGTCTATGTCCGGTCCCATG-3′	5′- GCTGATTTGTGGGTGTGGAA-3′
GAPDH	5′-GCGGGGGAGCAAAGGGT-3′	5′- TGGGTGGCAGTGATGGCATGG-3′

### Western blot

The protein extraction protocol from cancer cells and xenograft tumors was previously reported [31]. Briefly, the cells and tissues were treated with the RIPA lysis buffer (Thermo Fisher Scientific, USA) with a protease inhibitor cocktail (PIC, Invitrogen, USA). After centrifugation (12,000 rpm) for 30 min at 4℃, the supernatant was collected and applied to a 10% SDS-PAGE gel. After visualization by Ponceau S (Sigma, USA), the separated proteins were transferred to a PVDF membrane (Invitrogen, USA). Following block with 5% skim milk at 37 ℃ for 2 h and three 1 × PBS (pH 7.4) washes, the membrane was incubated with primary antibodies against RAC1 (1:1000, Abcam, USA), PAK1 (1:1000, CST, USA), p-PAK1 (1:2000, Abcam, USA), N-cadherin (1:1000, CST, USA), E-cadherin (1:2000, CST, USA), MMP2 (1:2000, CST, USA), MMP9 (1:2000, Abcam, USA), Vimentin (1:2000, Abcam, USA), and GAPDH (1:1000, CST, USA) at 4 ℃ for 24 h. Further, the membrane was treated with secondary antibodies (1:10,000, Jackson, USA) at 37 ℃ for 2 h. After three 1 × PBS washes, ECL reagents were applied to visualize the protein band (Amersham, UK). The grey values of protein bands were measured by the ImageJ software (National Institutes of Health, USA).

### CCK‑8 assay

Cell proliferation was examined by CCK-8 assay. Briefly, cells were transfected with indicated constructs for 72 h. The transfected cells were harvested, suspended, and seeded into 96‑well plates (1 × 10^4^ cells/well). After treatment at 0 h, 24 h, 48 h, and 72 h, 10 ul of CCK‑8 reagent (Thermo Fisher Scientific, USA) was added to each well followed by incubation at 37 ˚C for 2 h. Finally, the 450 nm absorbance was calculated by a microplate reader (BioRad Laboratories, USA).

### 5-ethynyl-2’-deoxyuridine (EdU) assay

EdU assay was adopted to determine cell viability. Briefly, following treatment with 200 μl of 5-ethynyl-20-deoxyuridine at room temperature for 2 h, the cells were fixed in 4% triformol for 30 min and were treated with 0.4% Triton X-100 for 10 min and 100 μl of Apollo reagent for 40 min. After staining by Hoechst (Sigma Aldrich, USA), the cell viability was determined according to the ratio between Hoechst-positive cells (blue) to EdU-positive cells (red).

### Colony formation assay

First, indicated constructs were transfected into cells for 6 h. Then the cells (1 × 10^4^ cells/well) were seeded into 6-well plates. The colonies were treated with 4% triformol (Invitrogen, USA) followed by staining with 0.1% crystal violet (Solarbio, Beijing, China). The cell colony numbers were counted under a stereomicroscope.

### Transwell assay

First, indicated constructs were transfected into cells for 72 h. Afterward, cells (1 × 10^4^ cells/well) were seeded into the top chamber of each well, and the RPMI‑1640 medium with 20% FBS was added to the low chamber. After 24 h incubation, the cells were fixed with 4% triformol (Invitrogen, USA) and stained using 1.5% crystal violet (Solarbio, Beijing, China) at 37 ℃.

### Flow cytometry analysis

Cell apoptosis was measured by Annexin V-FITC/PI Apoptosis Detection Reagent (Invitrogen, USA). The cells undergoing apoptosis were distinguished by a FACS flow cytometer (Beckman Coulter, USA) and counted with the FlowJo software (Ashland, USA).

### Luciferase reporter assay

The reporter vectors including the wild-type of circBRWD3(circBRWD3-WT) and RAC1 (RAC1-WT) as well as the mutant of circBRWD3 (circBRWD3-Mut) and RAC1 (RAC1-Mut) were cloned into pGL3-basic vector (Genechem, China), which were co-transfected with miR-142-3p mimics or miR-142-5p mimics into BC cell lines for 72 h. The luciferase activities (Promega, USA) were then calculated by a Luciferase Reporter Assay System (Ashland, USA).

### RNA pull-down assay

The RNA pull-down assay was performed to test the direct binding between circBRWD3/miR-142-3p and circBRWD3/miR-142-5p. Briefly, circBRWD3 probe or NC probe were transfected into BC cell lines by Lipofectamine™ 3000 (Invitrogen, USA) for 48 h. The transfected cells were incubated with magnetic beads (Life Technologies, USA). Following three PBS washes, the abundance of miR-142-3p or miR-142-5p transcripts was quantified by qRT-PCR.

### RNA immunoprecipitation (RIP) assay

The interaction between EIF4A3 and circBRWD3 in BC cells was investigated using the RNA immunoprecipitation assay. Briefly, cells were lysed by RIP lysis buffer for 45 min at 4℃. The magnetic beads conjugated with anti‑Ago2 (1:2000; Abcam; USA) or normal IgG (1:2000; Abcam; USA) were incubated with the cell extracts for 24 h at 4˚C. Then the beads were collected and treated with proteinase K. At last, circBRWD3 enrichment in immunoprecipitated RNAs was assessed by qRT-PCR.

### Xenograft Tumorigenesis

Nude mice were obtained from the SLAC Laboratory Animal Company (Shanghai, China). Animal administration was viewed and approved by the Ethics Committee of Shandong First Medical University. Sh-circBRWD3 or OE-circBRWD3 transfected BC cell lines (MDA-MB-231 and BT-549) (1 × 10^6^/mice) were injected subcutaneously into the mice. Tumor volume was measured weekly. After seven weeks, the mice were euthanized and tumor weights were documented.

### Statistical analysis

All data were analyzed and graphed in Prism 7.0 software (GraphPad Software, San Diego, CA, USA). The data were presented as mean ± SD. Two-tailed student’s *t*-test and one-way ANOVA were used to compare the data between two groups and among multiple groups, respectively. The survival curve was constructed by the Kaplan–Meier plot. *P* < 0.05 was considered statistically different. All experiments were repeated three times.

## Results

### CircBRWD3 is upregulated in breast cancer tissues and cell lines

**Fig. 1:**
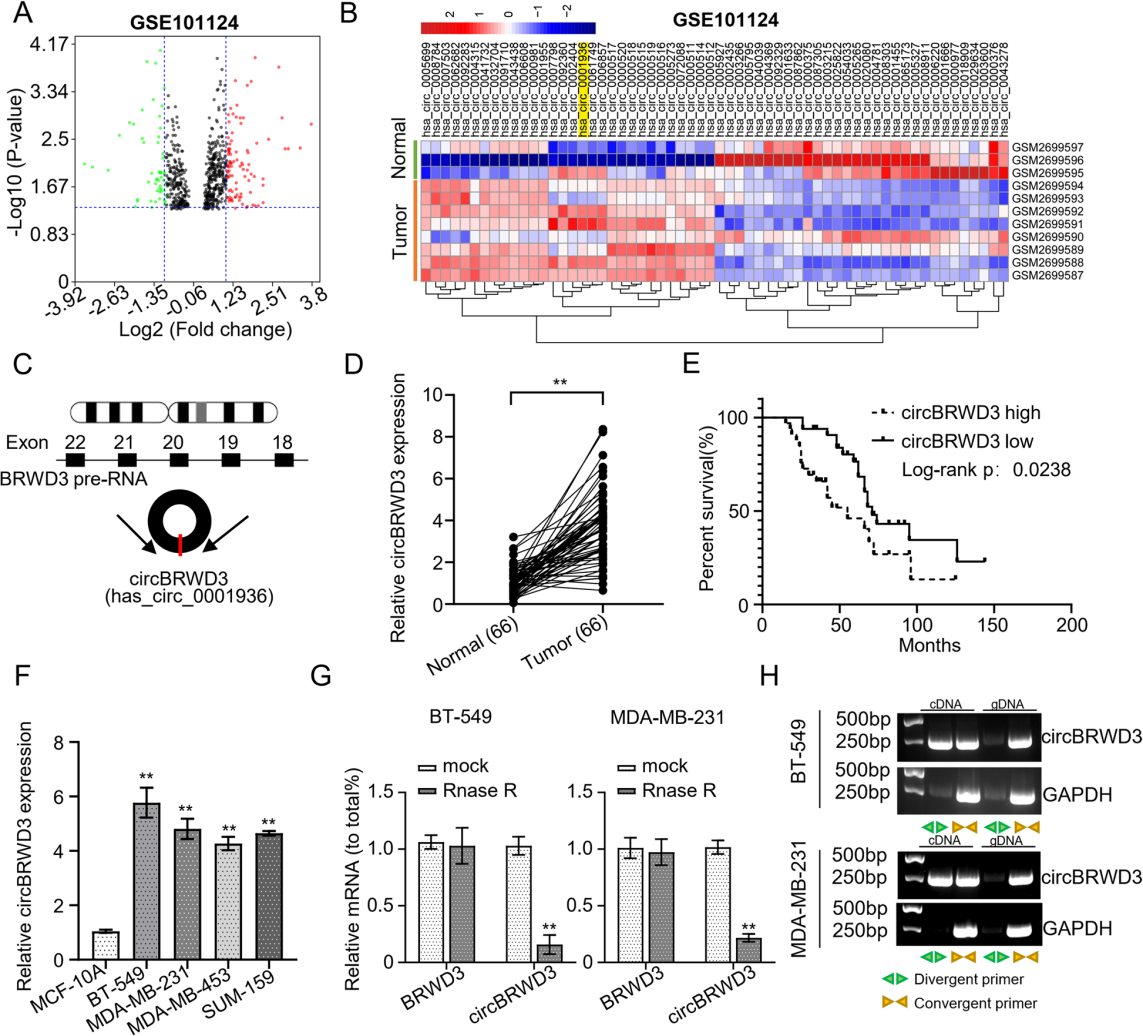
CircBRWD3 is upregulated in breast cancer tissues as well as cell lines. **A** The volcano plot of differentially expressed circRNAs in 5 pairs of normal mammary gland tissues and breast cancer tissues specimens based on the GEO database. **B** Clustered heatmap showing differentially expressed circRNAs in 5 pairs of normal mammary gland tissues and breast cancer tissues specimens in GEO database. **C** Schematic illustration of circBRWD3 formation by back splicing from the *BRWD3* gene. **D** The expression level of circBRWD3 in 66 pairs of breast cancer tissues and adjacent noncancerous specimens was calculated using qRT‐PCR. ^**^P < 0.01, Tumor vs. Normal. **E** Kaplan–Meier survival analyses of breast cancer patients with low or high circBRWD3 expression. **F** The expression level of circBRWD3 in four breast cancer cell lines (BT-549, MDA-MB-231, SUM-159, and MDA-MB-453) and the human normal breast epithelial cell line (MCF-10A) was quantified by qRT‐PCR. ^**^P < 0.01, MCF-10A vs. other cell lines. **G** The expression level of circBRWD3 and BRWD3 in breast cancer cell lines (MDA-MB-231 and BT-549) with or without RNase R treatment was calculated through qRT‐PCR. ^**^P < 0.01, mock vs. Rnase R in circBRWD3 group. **H** The existence of circBRWD3 was confirmed in breast cancer cell lines (MDA-MB-231 and BT-549). All experiments were done in triplicate

We reasoned that if circBRWD3 is involved in the BC progression, its expression should be dysregulated. To examine this speculation, we first downloaded the expression profiles of circRNAs from the GEO database and screened the dysregulated BC-relevant circRNAs. The screen revealed 151 dysregulated circRNAs (95 upregulated and 56 downregulated) in BC tissues (*n* = 5) compared with the normal mammary gland tissues (*n* = 5). Of these circRNAs, circBRWD3 was significantly upregulated in BC tissues (Fig. 1A & B). Then, we used a bioinformatics method to trace the origin of circBRWD3 and found that circBRWD3 was formed from exons 18 to 22 of the *BRWD3* gene (Fig. 1C). To validate the upregulation of circBRWD3 in BC, we determined the circBRWD3 transcript levels in 66 pairs of BC tissues and paired adjacent noncancerous specimens using qRT‐PCR analyses. CircBRWD3 expression levels were higher in BC tissues than in the adjacent noncancerous specimens (*p* < 0.01, Fig. 1D). Based on the median expression value of circBRWD3, we divided the 66 patients into low and high expression groups and assessed the correlation between the expression levels of circBRWD3 and the prognosis of patients. The Kaplan–Meier analysis revealed that the high expression levels of circBRWD3 were significantly correlated with the poor prognosis of patients (Fig. 1E). Further, using the chi-square test, we calculated the relationship between circBRWD3 expression and BC clinicopathological data and observed that the expression of circBRWD3 was significantly associated with the tumor size, TNM staging, and lymph node metastasis but not with the age and gender of patients as well as tumor differentiation (*p* < 0.01, Table 1). Moreover, echoing the tissue expression, circBRWD3was also strikingly upregulated in BC cell lines (MDA-MB-231, BT-549, SUM-159, and MDA-MB-453) (*p* < 0.01, Fig. 1F). Among the cell lines, BT-549 and MDA-MB-231 exhibited the most significant upregulation, which may reflect a higher efficiency of circBRWD3 knockdown. Therefore, we chose BT-549 and MDA-MB-231 for the following experiments. Next, we treated circBRWD3 with RNase R. We found that the linear BRWD3 was digested by RNase R, while the circBRWD3 was not, thereby proving the circular nature of circBRWD3 (*p* < 0.01, Fig. 1G). Additionally, we attempted to amplify circBRWD3 and BRWD3 from gDNA and cDNA and could only detect BRWD3 in the cDNA (Fig. 1H). Our results indicate that circBRWD3 is upregulated in BC, suggesting the involvement of circBRWD3 in BC progression.

### CircBRWD3 promotes breast cancer tumorigenesis in vitro

**Fig. 2:**
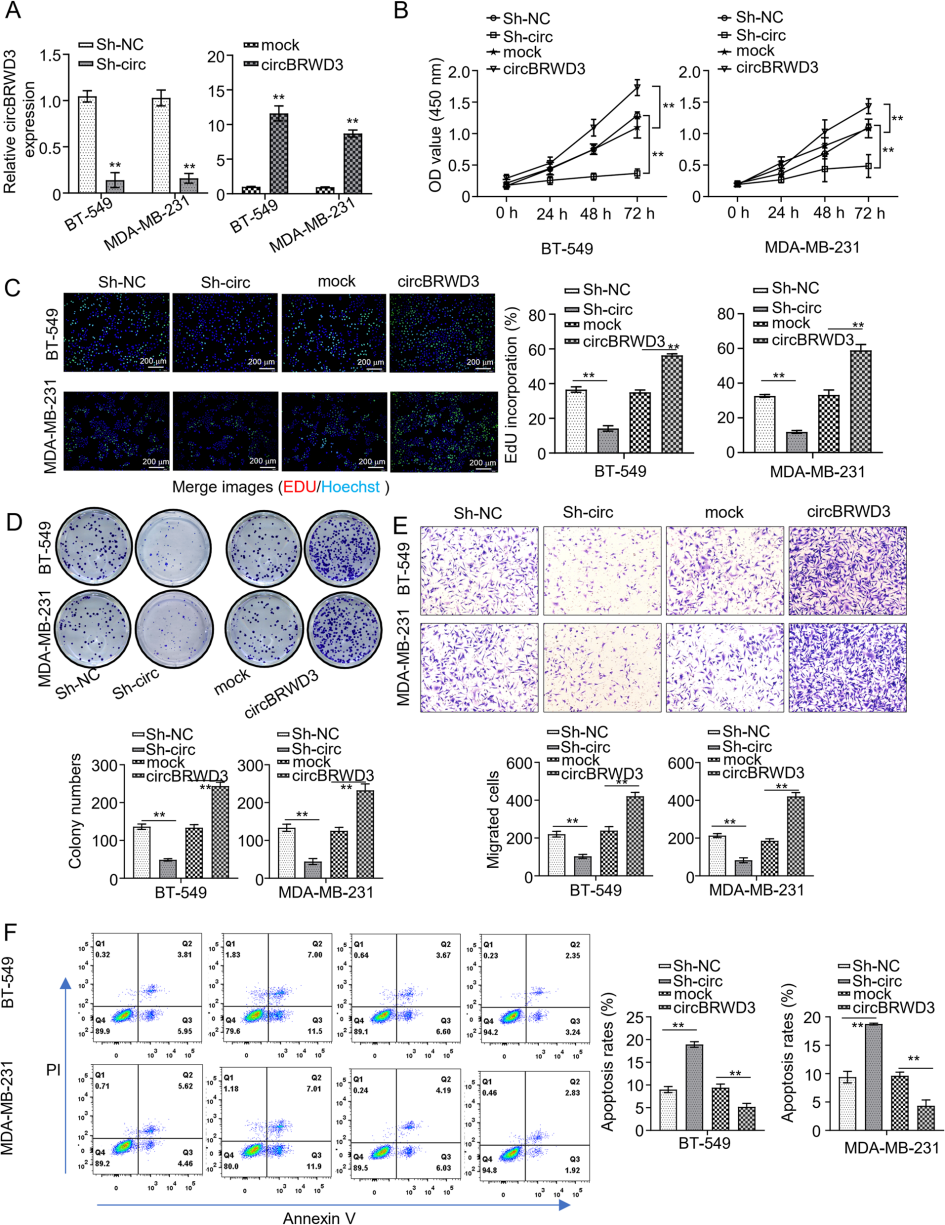
CircBRWD3 promotes breast cancer tumorigenesis in vitro. **A** The mRNA expression level of circBRWD3 in breast cancer cell lines (MDA-MB-231 as well as BT-549) transfected with OE-vector or OE-circBRWD3 plasmids and sh-NC or sh-circBRWD3 was calculated by qRT‐PCR. ^**^P < 0.01, Sh-NC vs. Sh-circ group; mock vs. circBRWD3 group. **B** The cell proliferation in (**A**) was determined using CCK-8 assay. ^**^P < 0.01, Sh-NC vs. Sh-circ group; mock vs. circBRWD3 group. **C** The cell viability in (**A**) was determined through EdU assay. ^**^P < 0.01, Sh-NC vs. Sh-circ group; mock vs. circBRWD3 group. **D** The colony number of cells in (**A**) was calculated using colony formation assay. ^**^P < 0.01, Sh-NC vs. Sh-circ group; mock vs. circBRWD3 group. **E** The cell migration capability in (**A**) was determined using transwell assay. ^**^P < 0.01, Sh-NC vs. Sh-circ group; mock vs. circBRWD3 group. **F**. The apoptosis of cells in (**A**) were assessed by flow cytometry analysis.^**^P < 0.01, Sh-NC vs. Sh-circ group; mock vs. circBRWD3 group. All experiments were carried out in triplicate. Sh-circ: sh-circBRWD3

Next, we set out to investigate the function of circBRWD3 using loss-and gain-of-function analysis. We knocked down or overexpressed circBRWD3 in MDA-MB-231 and BT-549 cell lines by transfection with sh-circBRWD3 or OE-circBRWD3, respectively (*p* < 0.01, Fig. 2A). Then, we used EdU, CCK-8, and colony formation assays to determine cell proliferation ability. The data elucidated that circBRWD3 knockdown repressed the proliferation of both cell lines manifested by diminished cell viability, EdU-positive cells, and reduced colony numbers, whereas circBRWD3 overexpression resulted in opposite phenotypes (*p* < 0.01, Fig. 2B-D). Meanwhile, we employed the transwell assay to determine cell metastasis ability. Both cell lines with circBRWD3 deficiency exhibited suppressed migration ability, while the ones overexpressing circBRWD3 showed enhanced migration ability (*p* < 0.01, Fig. 2E). Finally, we evaluated how the dysregulation of circBRWD3 affects the apoptosis of BC cells using the flow cytometry analysis. We found that circBRWD3 knockdown enhanced the apoptosis of MDA-MB-231 and BT-549 cells, whereas circBRWD3 overexpression suppressed the cell apoptosis (*p* < 0.01, Fig. 2F). In sum, these results suggest that circBRWD3 might act as an oncogene in the tumorigenesis of BC.

### EIF4A3 upregulates circBRWD3 in breast cancer cells

**Fig. 3:**
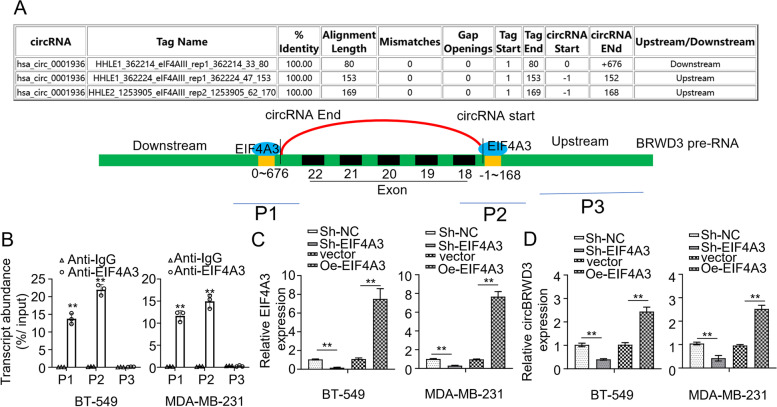
EIF4A3 upregulates circBRWD3 in breast cancer cells. **A** Schematic representation of the target region of EIF4A3 in the upstream region of circBRWD3 pre mRNA. **B** The relationship between EIF4A3 and circBRWD3 was confirmed by the RNA immunoprecipitation (RIP) assay. ^**^P < 0.01, Anti-IgG vs. Anti-EIF4A3 group. **C** The expression level of EIF4A3 in breast cancer cell lines (MDA-MB-231 as well as BT-549) transfected with OE-vector or OE-EIF4A3 plasmids and sh-NC or sh-EIF4A3 was determined using qRT‐PCR. ^**^P < 0.01, Sh-NC vs. Sh-EIF4A3 group; vector vs. Oe-EIF4A3 group. **D** The mRNA expression level of circBRWD3 in breast cancer cell lines (MDA-MB-231 as well as BT-549) transfected with OE-vector or OE-EIF4A3 plasmids and sh-NC or sh-EIF4A3 was determined using qRT‐PCR assay. ^**^P < 0.01, Sh-NC vs. Sh-EIF4A3 group; vector vs. Oe-EIF4A3 group. All experiments were carried out in triplicate. Oe-EIF4A3: EIF4A3 overexpression

Through screening the circular interactome database (https://circi-nteractome.nia.nih.gov/), we discovered a putative binding site for an RNA-binding protein EIF4A3 in the upstream region of the circBRWD3 precursor mRNA (pre-mRNA) (Fig. 3A). Then, we implemented the RIP assay to confirm the interaction between EIF4A3 and circBRWD3. We divided BRWD3 pre-mRNA into three segments, namely P1, P2, and P3. Of these, P1 and P2 spanning the binding site were effectively enriched in EIF4A3, while P3 that did contain the binding site was not (*p* < 0.01, Fig. 3B). Then, we studied the regulation of circBRWD3 expression by EIF4A3. To do this, we depleted and overexpressed EIF4A3 in MDA-MB-231 and BT-549 cell lines by transfection with sh-EIF4A3 and OE-EIF4A3 plasmids, respectively. We observed that the EIF4A3 transcript level was decreased in MDA-MB-231 and BT-549 cells transfected with sh-EIF4A3, while increased when transfected with the OE-EIF4A3, thereby confirming the efficiency of knockdown and overexpression (*p* < 0.01, Fig. 3C). Subsequently, we detected the change of circBRWD3 expression in the context of knowdown or overexpression of EIF4A. CircBRWD3 expression was evidently inhibited in both cell lines defective in sh-EIF4A3 but was enhanced in the cells overexpressing OE-EIF4A3 (*p* < 0.01, Fig. 3D). Together, our study indicates that EIF4A3 can facilitate circBRWD3 expression by binding to the upstream of BRWD3 pre-mRNA.

### CircBRWD3 functions as a sponge of miR-142-3p and miR-142-5p.

**Fig. 4:**
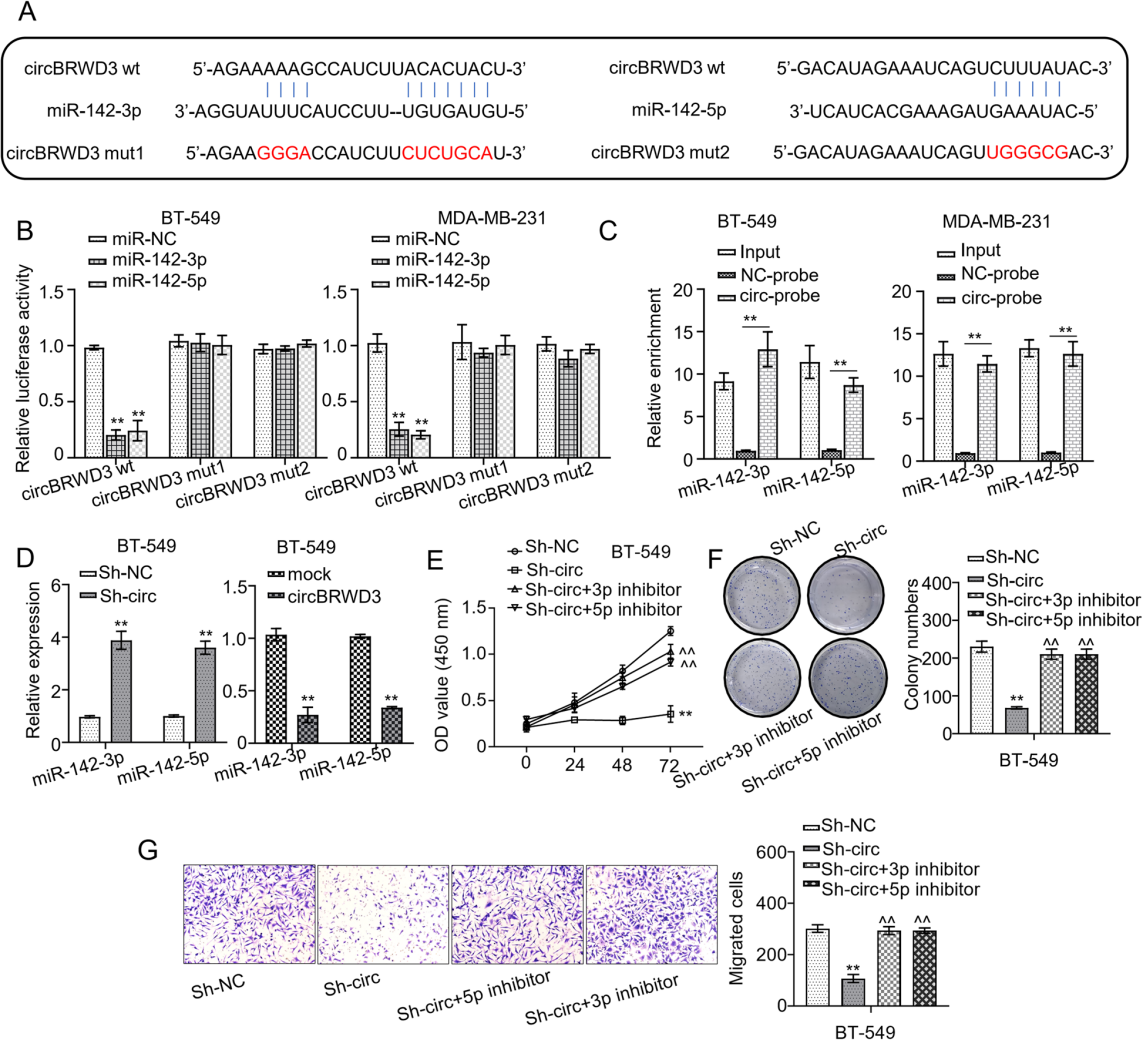
CircBRWD3 functions as a sponge of miR-142-3p and miR-142-5p. **A** Putative targets of wild-type and mutant circBRWD3 on miR-142-3p and miR-142-5p. **B** The relationship between circBRWD3 and miR-142-3p or circBRWD3 and miR-142-5p was calculated by luciferase reporter assay. ^**^P < 0.01, miR-NC vs. miR-142-3p/miR-142-5p group. **C** The relationship between circBRWD3 and miR-142-3p or circBRWD3 and miR-142-5p was calculated by RNA pull-down assay. ^**^P < 0.01, NC-probe vs. circ-probe group. **D** The expression levels of miR-142-3p and miR-142-5p in breast cancer cell lines (MDA-MB-231 as well as BT-549) transfected with OE-vector or OE-circBRWD3 plasmids and sh-NC or sh-circBRWD3 were calculated by qRT‐PCR. ^**^P < 0.01, Sh-NC vs. Sh-circ group; mock vs. circBRWD3 group. **E** The cell proliferation in breast cancer cell lines (MDA-MB-231 and BT-549) transfected with indicated constructs (sh-NC; sh-circBRWD3; sh-circBRWD3 + miR-142-3p inhibitor; sh-circBRWD3 + miR-142-5p inhibitor) were calculated using CCK-8 assay. ^**^P < 0.01, Sh-NC vs. Sh-circ group; ^^^^*P* < 0.01, Sh-circ vs. Sh-circ + 3p inhibitor/Sh-circ + 5p inhibitor. **F** The colony number of breast cancer cells (MDA-MB-231 and BT-549) transfected with indicated constructs (sh-NC; sh-circBRWD3; sh-circBRWD3 + miR-142-3p inhibitor; sh-circBRWD3 + miR-142-5p inhibitor) were calculated by colony formation assay. ^**^P < 0.01, Sh-NC vs. Sh-circ group; ^*^^*^*P* < 0.01, Sh-circ vs. Sh-circ + 3p inhibitor/Sh-circ + 5p inhibitor. **G** The cell migration in breast cancer cell lines (MDA-MB-231 and BT-549) transfected with indicated constructs (sh-NC; sh-circBRWD3; sh-circBRWD3 + miR-142-3p inhibitor; sh-circBRWD3 + miR-142-5p inhibitor) were calculated by transwell assay. ^**^P < 0.01, Sh-NC vs. Sh-circ group; ^^^^*P* < 0.01, Sh-circ vs. Sh-circ + 3p inhibitor/Sh-circ + 5p inhibitor. All experiments were carried out in triplicate. Sh-circ: sh-circBRWD3; circ-probe: circBRWD3-probe

Having proven the involvement of circBRWD3 BC tumorigenesis, we set out to molecularly dissect the underlying mechanism. First, we screened the circular interactome database (https://circ-interactome.nia-nih.gov/) to predict the downstream target of circBRWD3. The prediction revealed that circBRWD3 could bind to two miRNAs, miR-140-3p and miR-182 (Fig. 4A). Then, using the luciferase reporter and RNA pull-down assays we examined the direct binding between them. We found that co-transfection of WT-circBRWD3 with miR-142-3p mimics or miR-142-5p mimics reduced luciferase reporter activity in MDA-MB-231 and BT-549 cells, whereas the transfection of MUT-circBRWD3 with either of the miRNA mimics did not (*p* < 0.01, Fig. 4B). The RNA pull-down assay displayed that miR-142-3p and miR-142-5p were detected in the biotinylated circBRWD3 probe fraction, but not in the NC probe fraction (*p* < 0.01, Fig. 4C). Further, we examined how circBRWD3 affected miR-142-3p and miR-142-5p expression. We found that circBRWD3 deficiency increased the expression of these two microRNAs in MDA-MB-231 and BT-549 cell lines, whereas circBRWD3 overexpression decreased the levels of two miRNAs (*p* < 0.01, Fig. 4D). In addition, the suppressed cell proliferation of the two cell lines caused by circBRWD3 deficiency was relieved by the co-transfection with either of the miRNA inhibitors (*p* < 0.01, Fig. 4E-F). Similarly, the inhibition of the cell migrative ability of both BC cell lines conferred by circBRWD3 deficiency was reversed by the co-transfection with either of the miRNA inhibitors (*p* < 0.01, Fig. 4G). Collectively, these results support circBRWD3 as a sponge of both miR-142-3p and miR-142-5p to regulate breast cancer tumorigenesis.

### RAC1 is a direct target of miR-142-3p and miR-142-5p

**Fig. 5:**
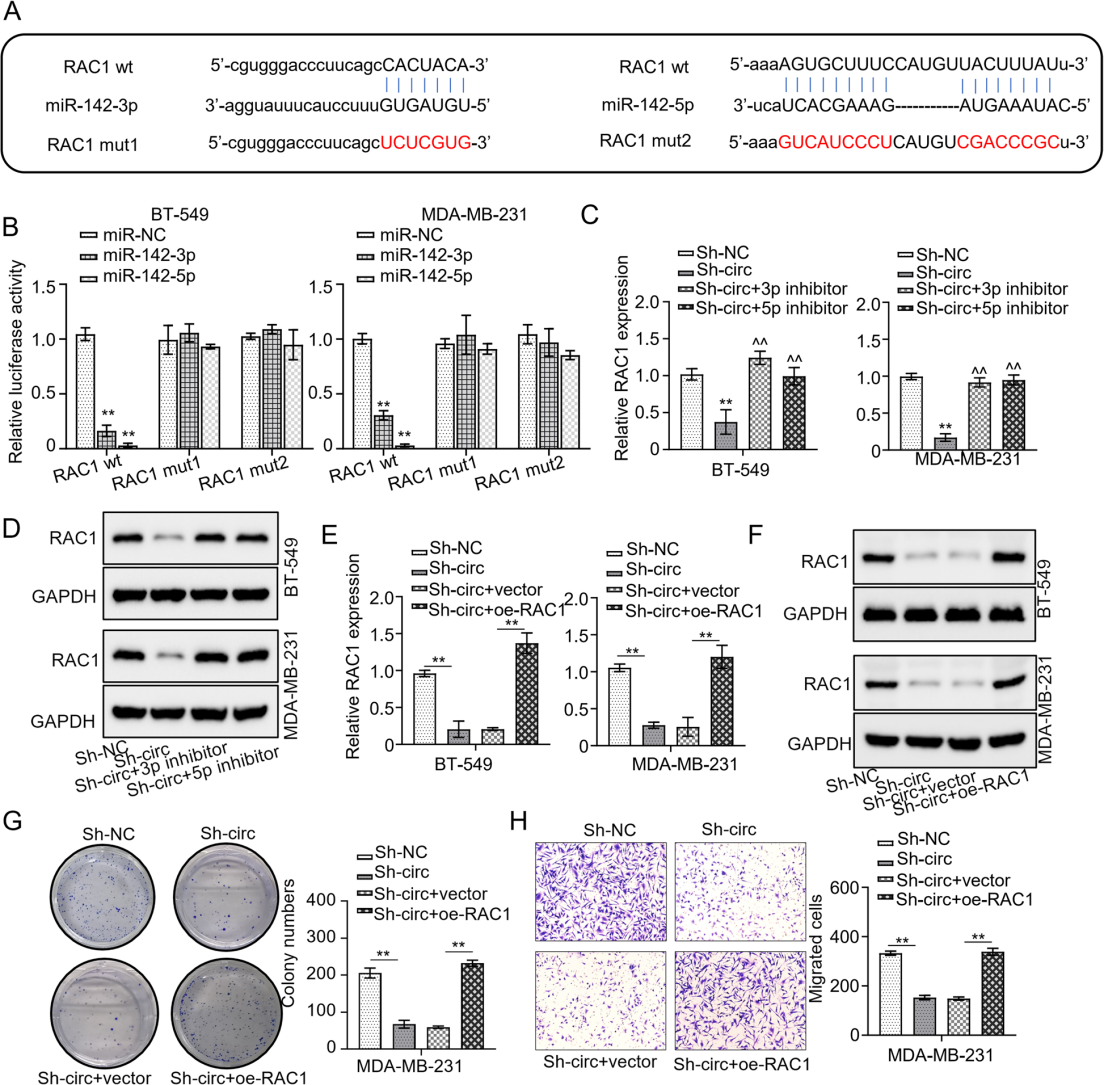
RAC1 is a direct target of miR-142-3p and miR-142-5p. **A** The complementary sequences of miR-142-3p and RAC1 or miR-142-5p and RAC1 were predicted by the STARBASE database. **B** The relationship between miR-142-3p and RAC1 or miR-142-5p and RAC1 was calculated by luciferase reporter assay. ^**^P < 0.01, miR-NC vs. miR-142-3p/miR-142-5p group. **C** The mRNA expression levels of RAC1 in breast cancer cell lines (MDA-MB-231 and BT-549) transfected with indicated constructs (sh-NC; sh-circBRWD3; sh-circBRWD3 + miR-142-3p inhibitor; sh-circBRWD3 + miR-142-5p inhibitor) were determined by qRT‐PCR. ^**^P < 0.01, Sh-NC vs. Sh-circ group; ^^^^*P* < 0.01, Sh-circ vs. Sh-circ + 3p inhibitor/Sh-circ + 5p inhibitor. **D** The protein levels of RAC1 in breast cancer cell lines (MDA-MB-231 as well as BT-549) transfected with indicated constructs (sh-NC; sh-circBRWD3; sh-circBRWD3 + miR-142-3p inhibitor; sh-circBRWD3 + miR-142-5p inhibitor) were calculated by western blot. **E** The mRNA expression levels of RAC1 in breast cancer cell lines (MDA-MB-231 and BT-549) transfected with indicated constructs (sh-NC; sh-circBRWD3; sh-circBRWD3 + OE-vector; sh-circBRWD3 + OE-RAC1) were assessed by qRT‐PCR. ^**^P < 0.01, Sh-NC vs. Sh-circ group; Sh-circ + vector vs. Sh-circ + oe-RAC1. **F** The protein levels of RAC1 in breast cancer cell lines (MDA-MB-231 and BT-549) transfected with indicated constructs (sh-NC; sh-circBRWD3; sh-circBRWD3 + OE-vector; sh-circBRWD3 + OE-RAC1) were determined using western blot. **G** The colony numbers of breast cancer cells (MDA-MB-231 as well as BT-549) transfected with indicated constructs (sh-NC; sh-circBRWD3; sh-circBRWD3 + OE-vector; sh-circBRWD3 + OE-RAC1) were calculated by colony formation assay. ^**^P < 0.01, Sh-NC vs. Sh-circ group; Sh-circ + vector vs. Sh-circ + oe-RAC1. **H** The cell migration in breast cancer cell lines (MDA-MB-231 and BT-549) transfected with indicated constructs (sh-NC; sh-circBRWD3; sh-circBRWD3 + OE-vector; sh-circBRWD3 + OE-RAC1) was examined by transwell assay. ^**^P < 0.01, Sh-NC vs. Sh-circ group; Sh-circ + vector vs. Sh-circ + oe-RAC1. All experiments were carried out in triplicate. Sh-circ: sh-circBRWD3; Oe-EIF4A3: EIF4A3 overexpression

To identify the downstream target of miR-142-3p and miR-142-5p, we first screened the candidate(s) through the STARBASE database, the screening revealed that RAC1 was a potential target gene of the two miRNAs (Fig. 5A). The luciferase reporter assay displayed that both MDA-MB-231 and BT-549 cells co-transfected with WT-RAC1 together with either miR-142-3p mimics or miR-142-5p mimics showed reduced luciferase reporter activity, but the cells transfected with MUT-RAC1 and either of the miRNA mimics exhibited strong activity (*p* < 0.01, Fig. 5B). Further, both the mRNA and protein levels of RAC1 were diminished in MDA-MB-231 BT-549 cell lines transfected with sh-circBRWD3, an effect that was reversed by co-transfection of sh-circBRWD3 with either of the miRNA inhibitors (*p* < 0.01, Fig. 5C-D). Moreover, both transcript and protein levels of RAC1 were significantly decreased in both cell lines transfected with sh-circBRWD3 but were not in the cells transfected with sh-circBRWD3 and OE-RAC1 (*p* < 0.01, Fig. 5E-F). Other than that, the impaired cell proliferation caused by circBRWD3 deficiency could be rescued by the overexpression of RAC1 (*p* < 0.01, Fig. 5G). Similarly, the suppression of cell migration ability conferred by circBRWD3 was reversed by the overexpression of RAC1 (*p* < 0.01, Fig. 5H). Together, these data demonstrate that circBRWD3 regulates RAC1 expression by competitively binding to miR-142-3p and miR-142-5p.

### CircBRWD3 facilitates breast cancer progression in vivo

**Fig. 6:**
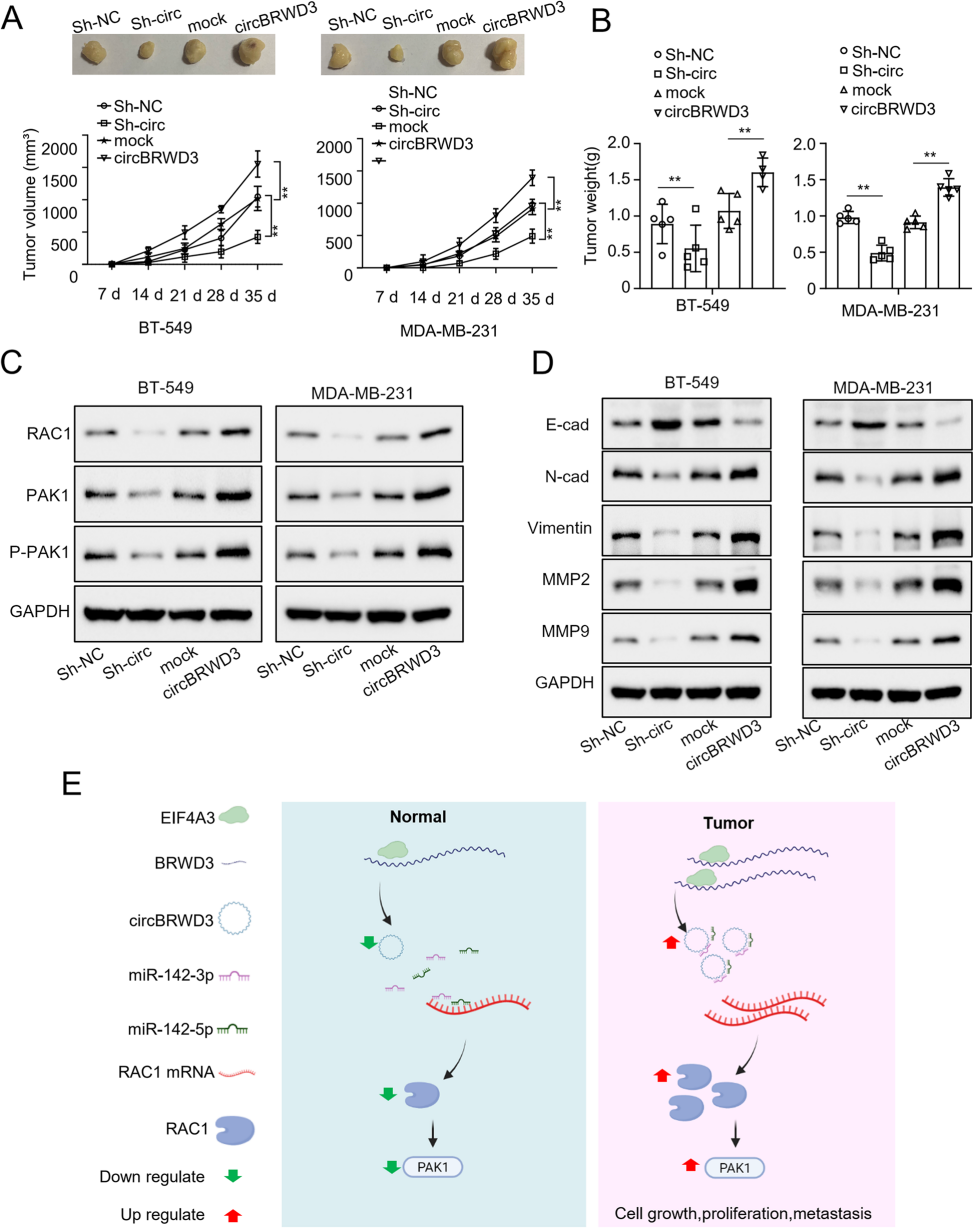
CircBRWD3 promotes tumorigenesis of breast cancer via miR-142/RAC1-PAK1 signaling. **A** Nude mice were subcutaneously injected with the breast cancer cells transfected with indicated constructs (sh-NC; sh-circBRWD3; OE-vector; OE-circBRWD3). After 35 days, the animals were sacrificed and the tumor volume was measured. ^**^P < 0.01, Sh-NC vs. Sh-circ group; mock vs. circBRWD3 group. **B** Nude mice were subcutaneously injected with the breast cancer cell transfected with indicated constructs (sh-NC; sh-circBRWD3; OE-vector; OE-circBRWD3). After 35 days, the animals were sacrificed and the tumors was weighted. ^**^P < 0.01, Sh-NC vs. Sh-circ group; mock vs. circBRWD3 group. **C** The protein levels of RAC1, PAK1, and p-PAK1 in (**B**) were determined using western blot. **D** The protein levels of E-cadherin, N-cadherin, Vimentin, and MMP9 in (**B**) were determined using western blot. **E** Schematic representation of the molecular mechanism of EIF4A3/circBRWD3/ miR-142-3p_miR-142-5p /RAC1/PAK1 axis in breast cancer. All experiments were carried out in triplicate. Sh-circ: sh-circBRWD3; E-cad: E-cadherin; N-cad: N-cadherin

To verify the function of circBRWD3 in vivo, we established a mice BC model by subcutaneously injecting nude mice with MDA-MB-231 and BT-549 cells transfected with either sh-circBRWD3 or OE-circBRWD3. Tumor volumes were measured every 7 days post-injection. After 35 days, the mice were euthanized, and the tumors were dissected and weighted. CircBRWD3 knockdown impaired tumor growth as reflected by light tumor weight, while circBRWD3 overexpression facilitated the tumor growth, resulting in heavy tumor weight (*p* < 0.01, Fig. 6A-B).

### CircBRWD3 promotes breast cancer tumorigenesis via miR-142-3p_miR-142-5p/RAC1/PAK1 signaling

SinceRAC1 expression was subjected to regulation by circBRWD3, we tested whether circBRWD3 promoted tumorigenesis by regulating RAC/PAK1 signaling. To do this, we took advantage of the in vivo tumor model we established. We collected the xenograft tumor tissues from the mice and quantified the levels of the key molecular components of the RAC/PAK1 signaling. In the xenograft tumor tissue overexpressing circBRWD3, RAC1, PAK1, and p- PAK1 were dramatically upregulated, while in the tissue with circBRWD3 knockdown, they were significantly downregulated (Fig. 6C). Moreover, in the circBRWD3 overexpression tumor model, E-cadherin was upregulated, while N-cadherin, Vimentin, MMP2, and MMP9 were downregulated. By contrast, the circBRWD3 knockdown tumor model exhibited opposite phenotypes with a decreased level of E-cadherin and increased levels of N-cadherin, Vimentin, MMP2, and MMP9 (Fig. 6D). Collectively, our work suggests that the RAC/PAK1 signaling is positively regulated by circBRWD3, which is conducive to BC progression (Fig. 6E).

## Discussion

It is well accepted that merely around 12% of the genomic sequences are encoding proteins, while most of the others are found to encode ncRNAs that typically act as potent regulators of gene expression [32]. Over past decades, mounting evidence has demonstrated that dysregulated ncRNAs are closely associated with malignant tumor tumorigenesis and progression [33]. Compared to long non-coding RNAs and miRNAs, circRNAs garner greater interest in the ncRNA research field, especially in tumorigenesis and malignant tumor development [34]. Moreover, on account of their unique molecular structures and cell/tissue-specific expressions, circRNAs are considered as better therapeutic targets and early diagnostic biomarkers for malignant tumors than linear mRNAs [7, 35]. However, the role of circRNAs in BC tumorigenesis has been underappreciated with a large number of circRNAs understudied.

Here, we focused on the function and mechanism of a newly discovered circRNA, namely circBRWD3. It was dramatically upregulated in BC tissues, which predicts a poor prognosis. CircBRWD3 deficiency repressed cell proliferation and metastasis and promoted cell apoptosis; however, circBRWD3 overexpression resulted in the opposite phenotype. Mechanistically, EIF4A3 could facilitate circBRWD3 expression through targeting the upstream of BRWD3 pre-mRNA. Additionally, circBRWD3 sponged miR-142-3p and miR-142-5p to regulate the expression of RAC1, thereby activating RAC1/PAK1 signaling pathway and facilitating the tumorigenesis and progression of BC.

CircRNAs typically act as ceRNAs to direct target miRNAs to regulate gene expression [36, 37], which significantly advance the progression of diverse malignant tumors, including BC. For instance, circATXN7 promotes BC tumorigenesis by regulating miR-149-5p expression [38]. CircKDM4B represses BC progression through sponging miR-675 [39]. Circ_0008500 facilitates tumorigenesis and promotes radiosensitivity in BC via inhibiting miR-758-3p expression [40]. In our study, we found that circBRWD3 directly targeted miR-142-3p and miR-142-5p. In addition, circBRWD3 deficiency repressed cell proliferation and metastasis, which was rescued by co-transfection with either of the miRNA inhibitors, suggesting that circBRWD3 regulates the BC tumorigenesis through sponging miR-142-3p and miR-142-5p.

Then, what is the target gene of these two miRNAs? First, through screening the STARBASE database, we found that RAC1 was the potential target gene. Next, we confirmed RAC1 as the direct target gene using luciferase report assay, qRT‐PCR quantification, and western blot analyses. RAC1 can regulate many cellular activities, such as cell proliferation, metastasis, and epithelial-mesenchymal transition [26]. Due to the important regulatory roles, RAC1 has been proposed as one of the etiological factors in multiple malignant tumors, including BC [27–29]. In the current study, we found that whencircBRWD3 expression was inhibited, the cell proliferation and metastasis of cancer were suppressed, a phenomenon that could be reversed by RAC1 overexpression. RAC1 is a kind of GTPase, modulating diverse cellular processes via serine/threonine kinases (PAKs) [41]. PAK1 is the most common subtype of PAKs and has been found dysregulated in malignant tumors. It has been observed that the abnormal expression of PAK1 is important for the tumorigenesis of diverse malignant tumors [42]. RAC1/PAK1 signaling pathway has been implicated in BC progression. For instance, RAC1/PAK1 signaling has been implicated in the tamoxifen resistance of BC cells [43] and can regulate the metastasis and angiogenesis of BC [29]. Consistent with these studies, our study elucidates that the RAC1/PAK1 signaling pathway is subjected to the regulation by miR-142-3p and miR-142-5p and is involved in the tumorigenesis of BC. EIF4A3 is an RNA binding protein and is a key constituent of the exon junction complex, acting as a vital post-transcriptional regulator for mRNA transport, splicing, surveillance, and translation [44]. EIF4A3 can target the upstream region of a circRNA pre mRNA to regulate its expression [45]. Consistent with the reported function, we found that EIF4A3c can bind to the upstream region of circBRWD3 pre mRNA and positively regulates its expression. Whether EIF4A3c recruits other factors to form a regulatory complex to exert its function is an open question and worth further studies.

## Conclusion

In conclusion, we elucidated the function of circBRWD3 in BC progression and delineates a new regulatory axis consisting of EIF4A3/circBRWD3/miR-142-3p_miR-142-5p/RAC1/PAK1, providing new therapeutic targets for drug design and early diagnosis.

## Supplementary Information

**Additional file** **1.**

## Data Availability

The datasets used and/or analyzed during the current study are available from the corresponding author on reasonable request.
