# Supplementary material for: EIF4A3-induced circBRWD3 promotes tumorigenesis of breast cancer through miR-142-3p_miR-142-5p/RAC1/PAK1 signaling
Source: BMC Cancer. 2022 Nov 28;22:1225. doi: 10.1186/s12885-022-10200-7 (PMC9703775; doi:10.1186/s12885-022-10200-7)
Supplement: Supplementary file 1 — Additional file 1. [file 12885_2022_10200_MOESM1_ESM.pptx]

## Slide 1
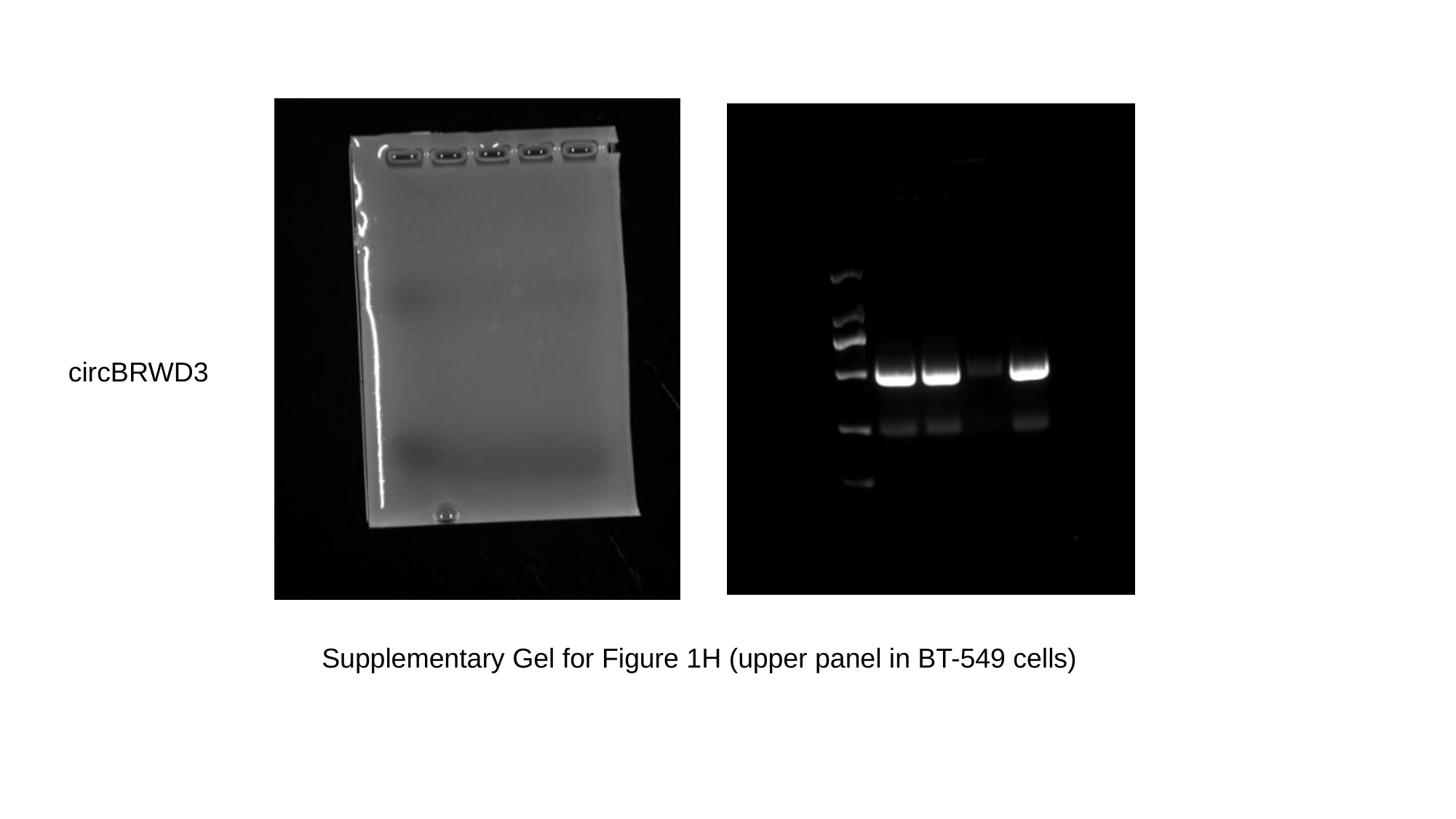

circBRWD3
Supplementary Gel for Figure 1H (upper panel in BT-549 cells)

## Slide 2
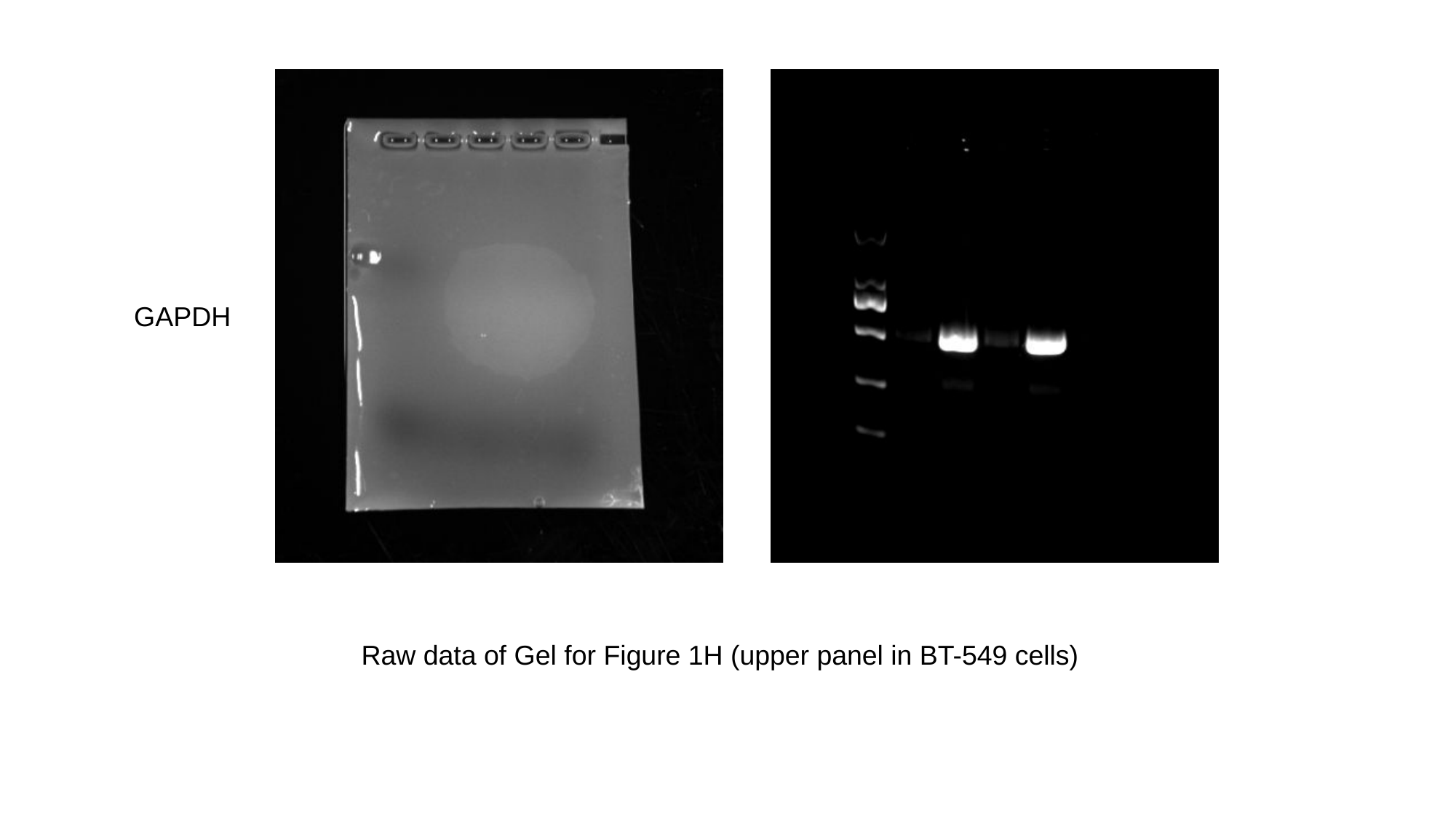

GAPDH
Raw data of Gel for Figure 1H (upper panel in BT-549 cells)

## Slide 3
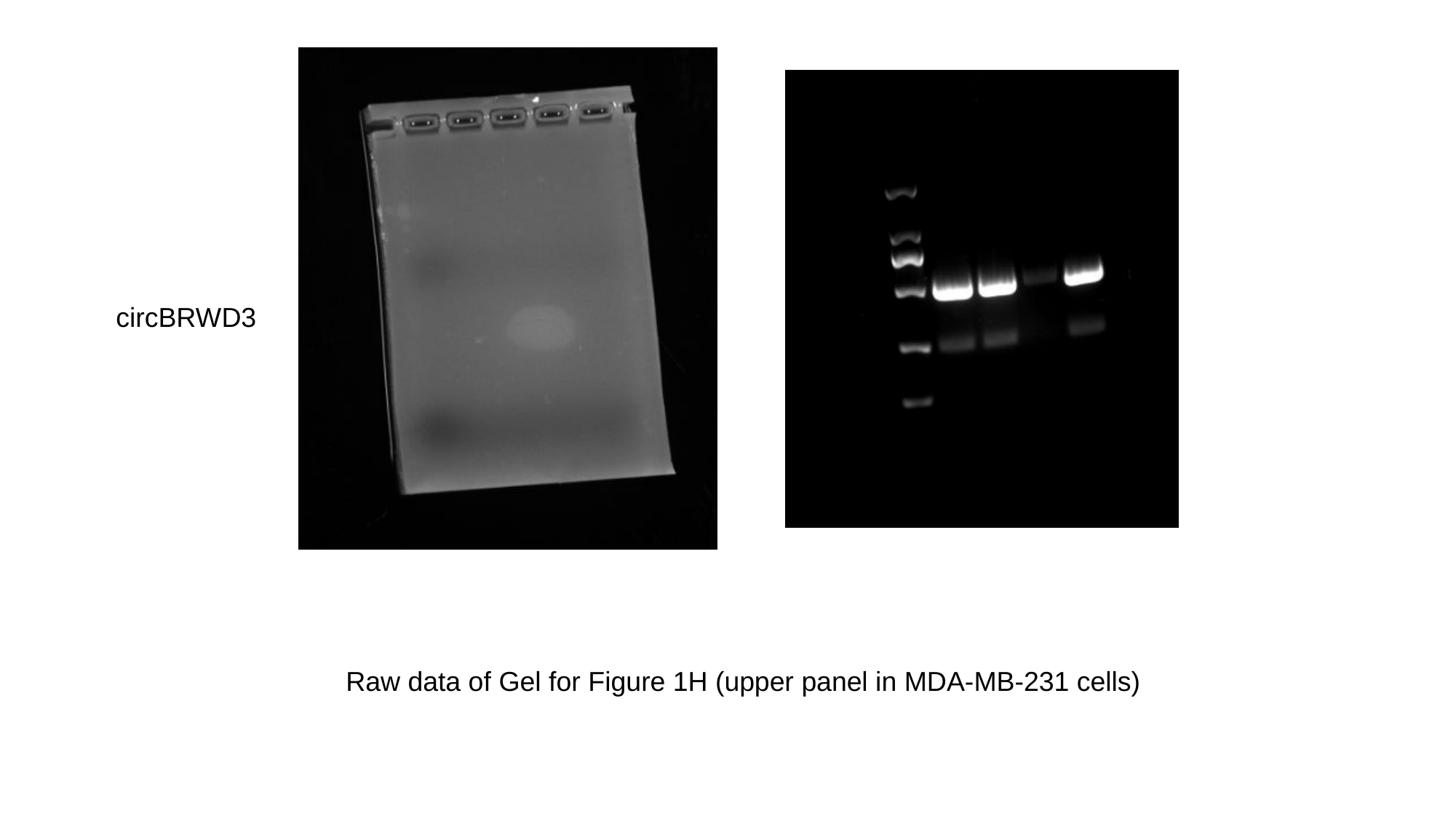

circBRWD3
Raw data of Gel for Figure 1H (upper panel in MDA-MB-231 cells)

## Slide 4
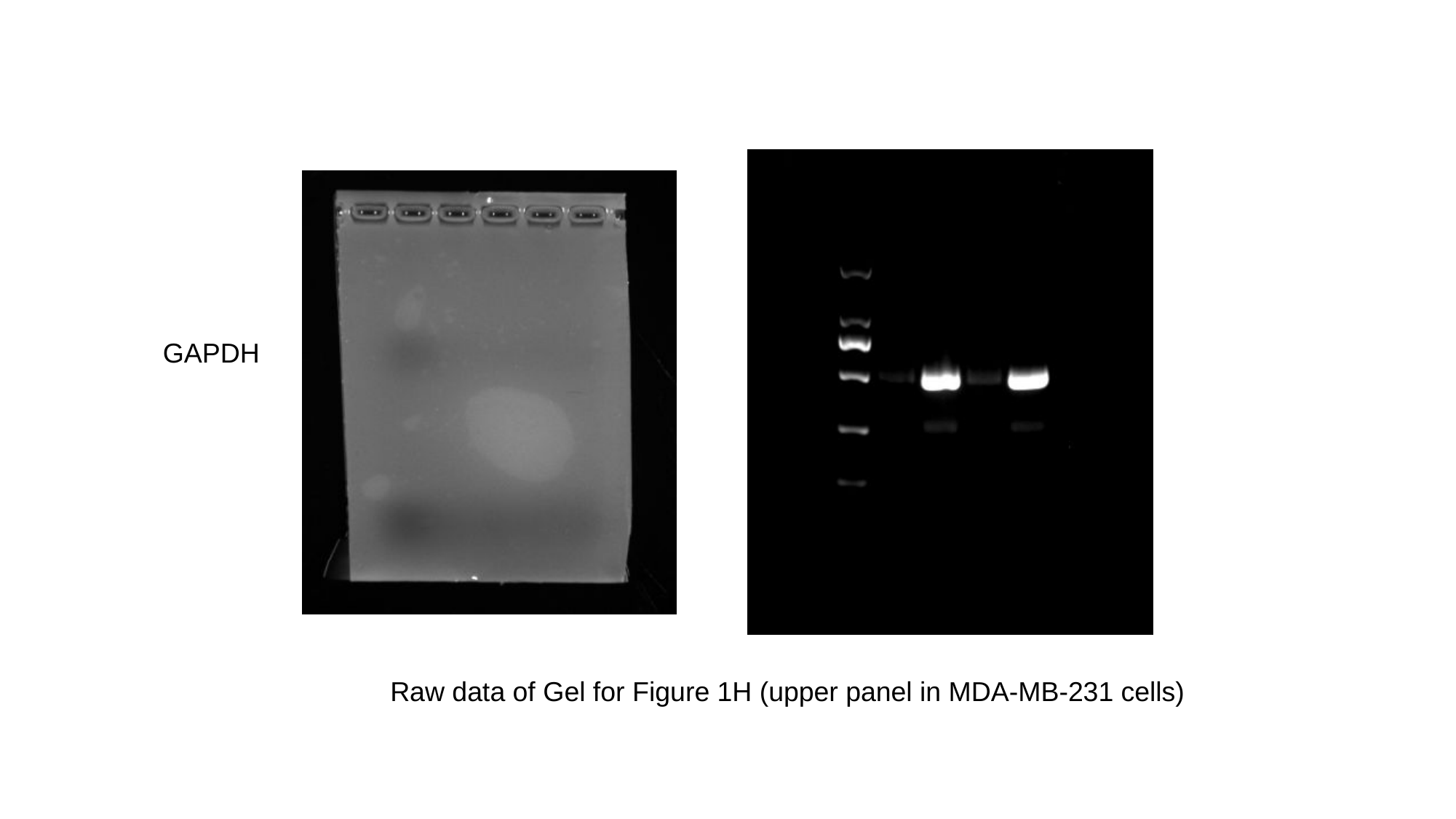

GAPDH
Raw data of Gel for Figure 1H (upper panel in MDA-MB-231 cells)

## Slide 5
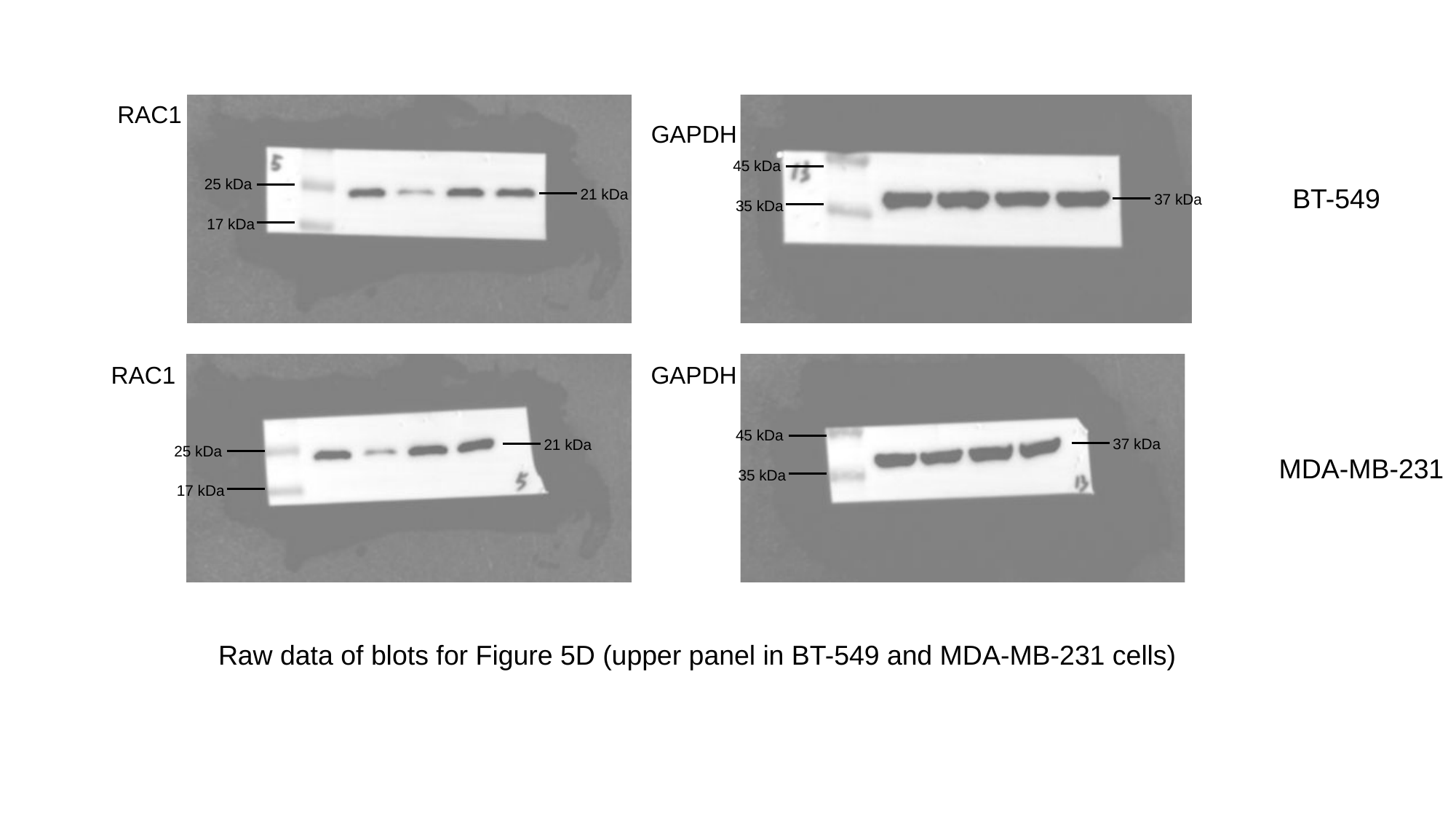

RAC1
GAPDH
45 kDa
25 kDa
BT-549
21 kDa
37 kDa
35 kDa
17 kDa
RAC1
GAPDH
45 kDa
37 kDa
21 kDa
25 kDa
MDA-MB-231
35 kDa
17 kDa
Raw data of blots for Figure 5D (upper panel in BT-549 and MDA-MB-231 cells)

## Slide 6
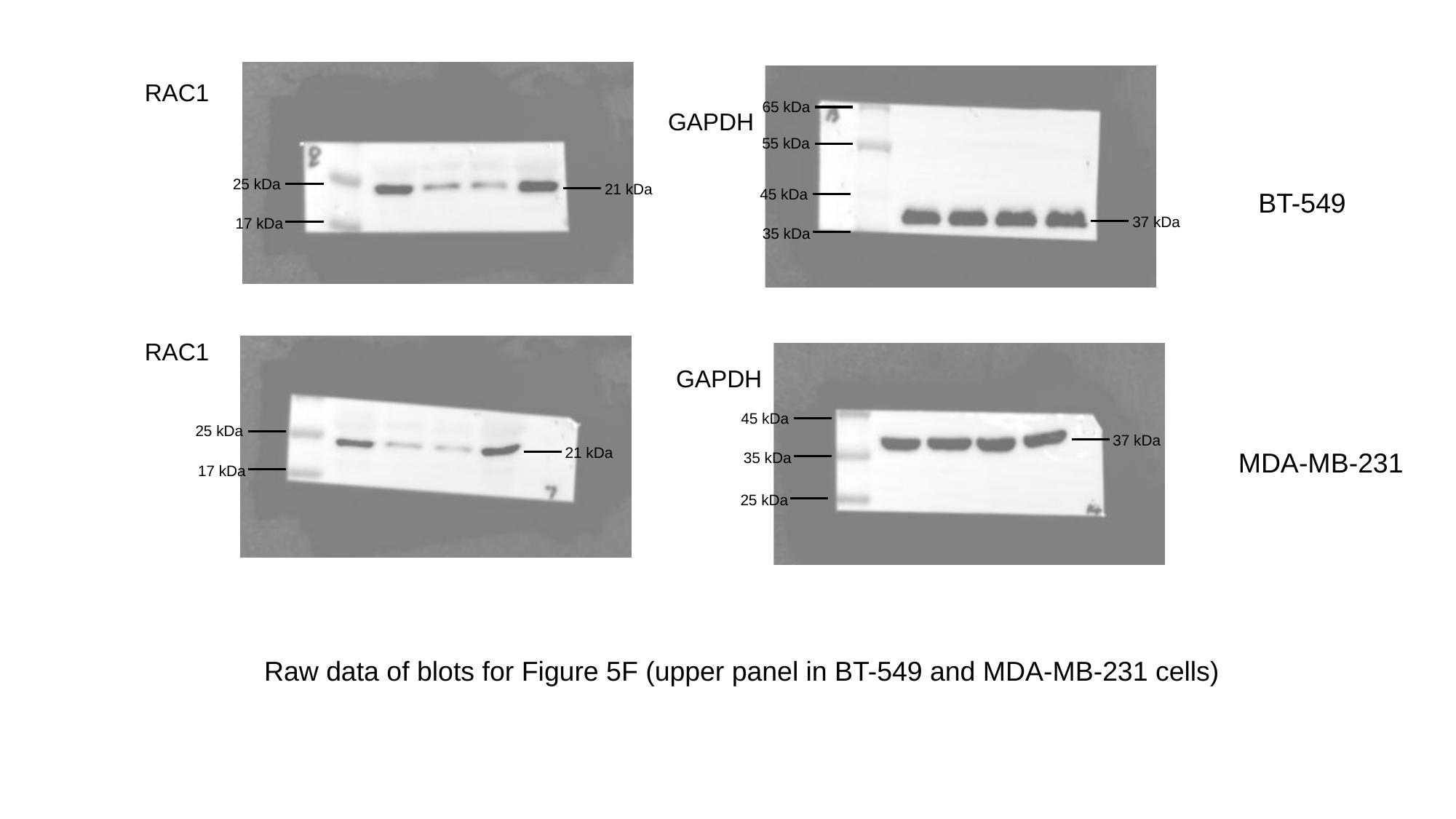

RAC1
65 kDa
GAPDH
55 kDa
25 kDa
21 kDa
45 kDa
BT-549
37 kDa
17 kDa
35 kDa
RAC1
GAPDH
45 kDa
25 kDa
37 kDa
21 kDa
MDA-MB-231
35 kDa
17 kDa
25 kDa
Raw data of blots for Figure 5F (upper panel in BT-549 and MDA-MB-231 cells)

## Slide 7
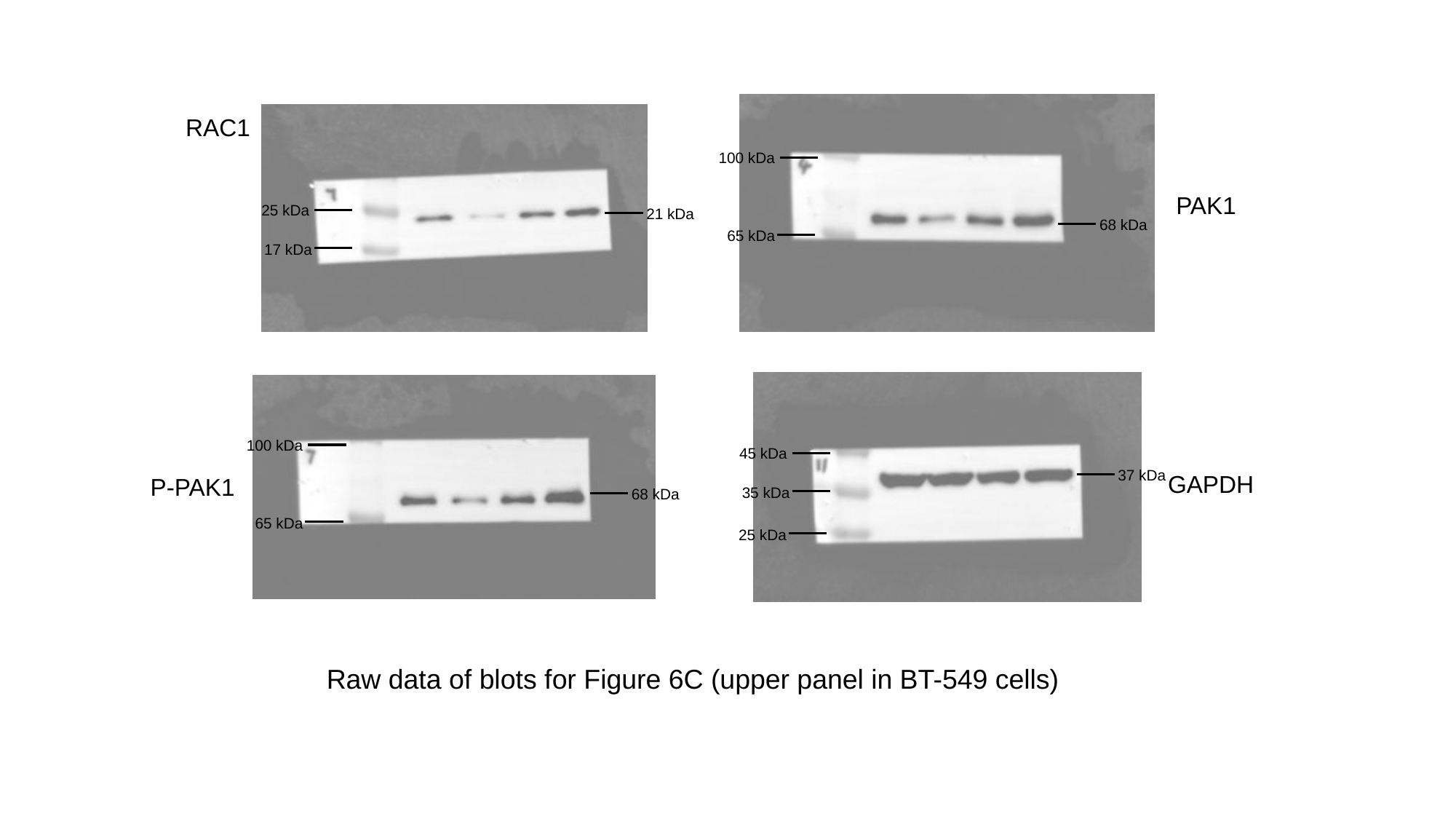

RAC1
100 kDa
PAK1
25 kDa
21 kDa
68 kDa
65 kDa
17 kDa
100 kDa
45 kDa
37 kDa
GAPDH
P-PAK1
35 kDa
68 kDa
65 kDa
25 kDa
Raw data of blots for Figure 6C (upper panel in BT-549 cells)

## Slide 8
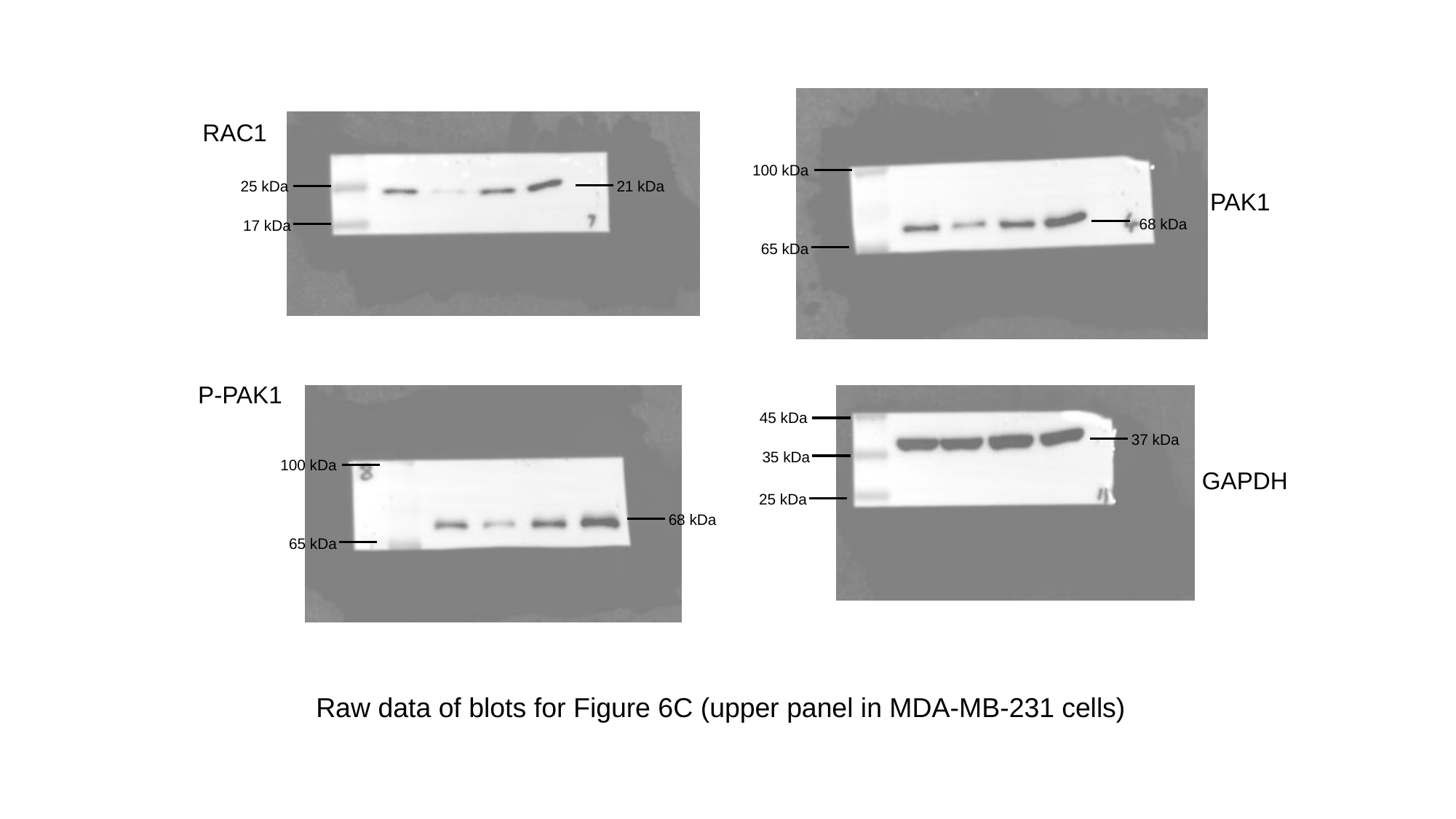

RAC1
100 kDa
25 kDa
21 kDa
PAK1
68 kDa
17 kDa
65 kDa
P-PAK1
45 kDa
37 kDa
35 kDa
100 kDa
GAPDH
25 kDa
68 kDa
65 kDa
Raw data of blots for Figure 6C (upper panel in MDA-MB-231 cells)

## Slide 9
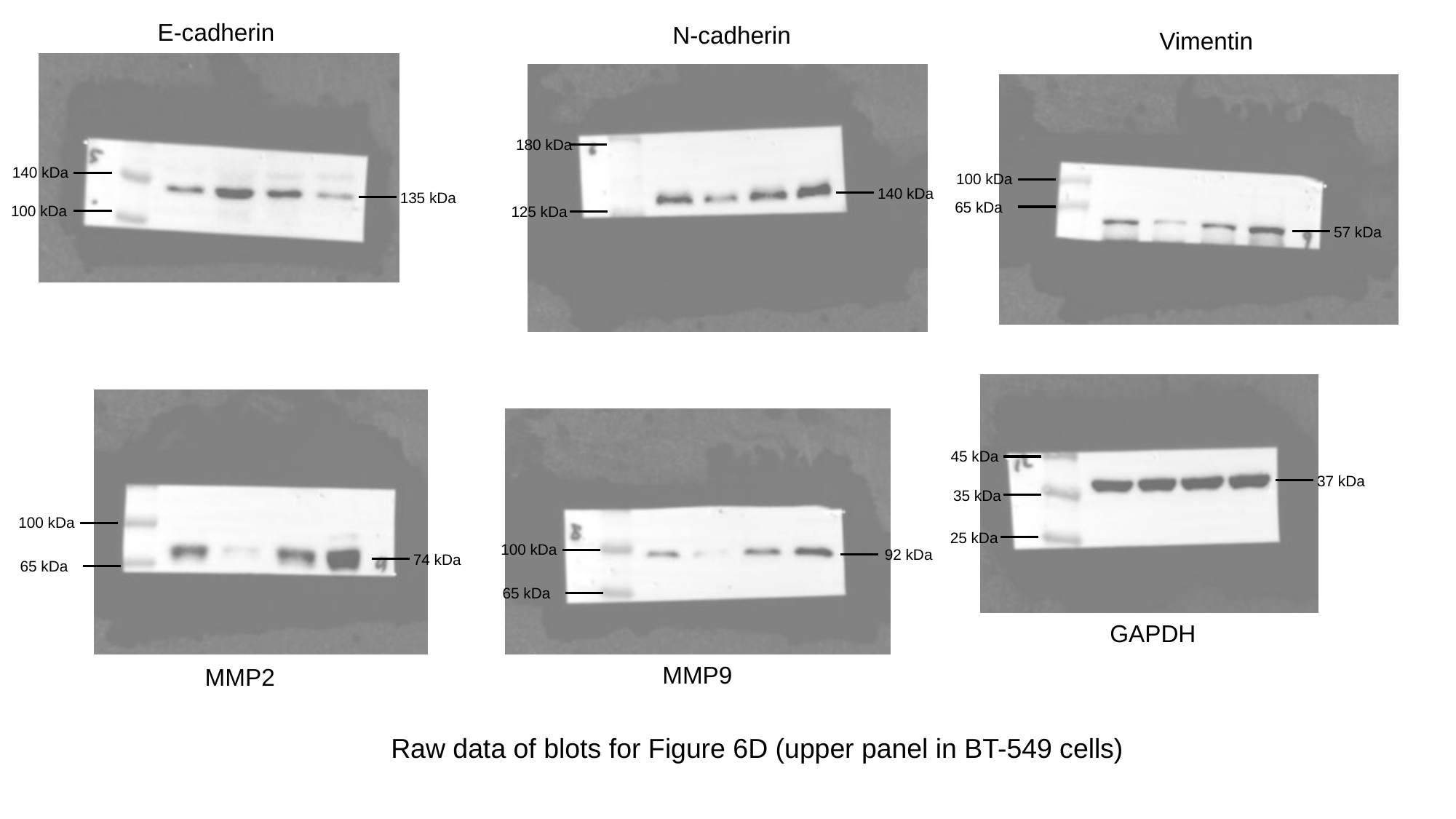

E-cadherin
N-cadherin
Vimentin
180 kDa
140 kDa
100 kDa
140 kDa
135 kDa
65 kDa
100 kDa
125 kDa
57 kDa
45 kDa
37 kDa
35 kDa
100 kDa
25 kDa
100 kDa
92 kDa
74 kDa
65 kDa
65 kDa
GAPDH
MMP9
MMP2
Raw data of blots for Figure 6D (upper panel in BT-549 cells)

## Slide 10
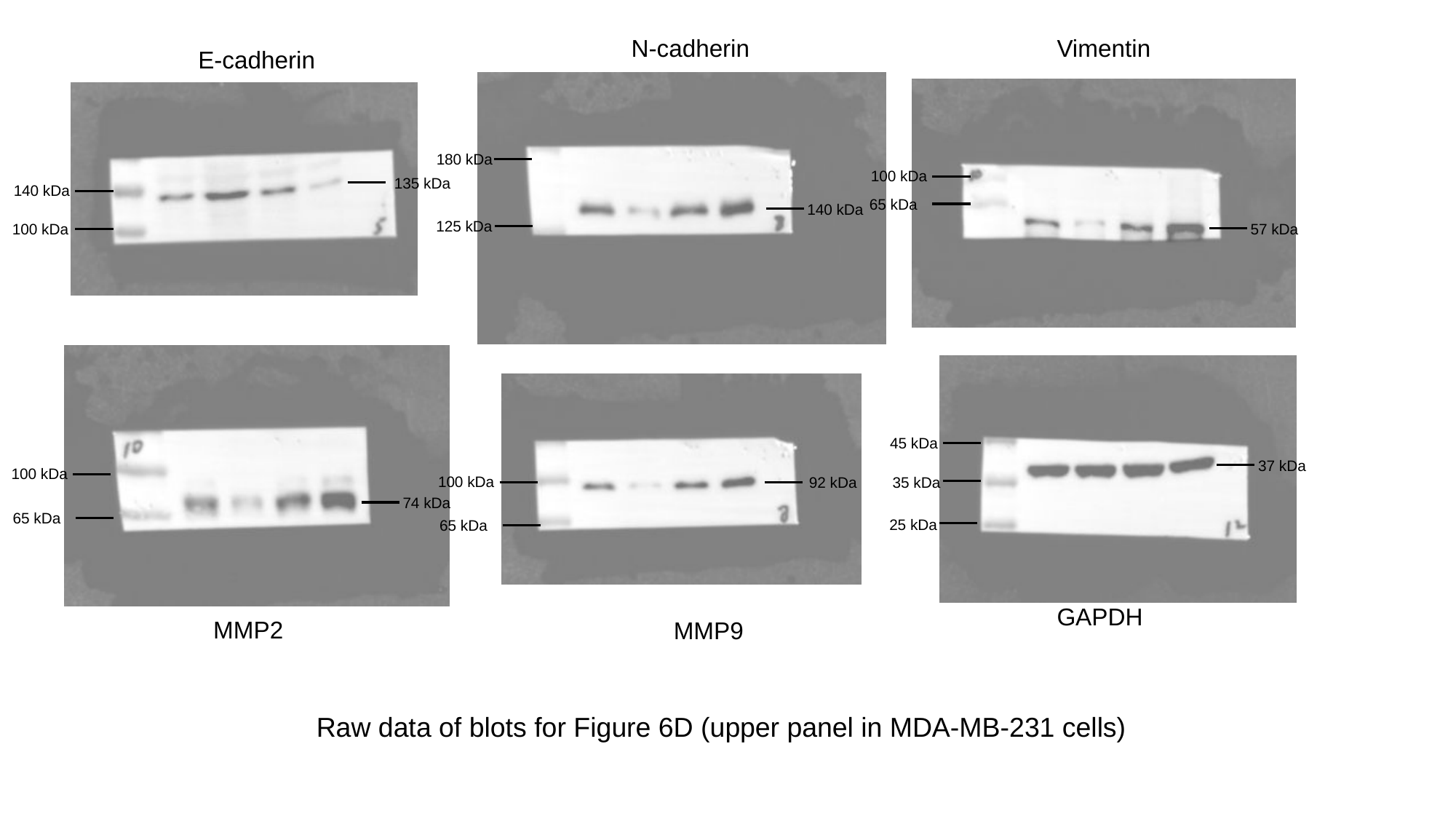

Vimentin
N-cadherin
E-cadherin
180 kDa
100 kDa
135 kDa
140 kDa
65 kDa
140 kDa
125 kDa
100 kDa
57 kDa
45 kDa
37 kDa
100 kDa
100 kDa
35 kDa
92 kDa
74 kDa
65 kDa
25 kDa
65 kDa
GAPDH
MMP2
MMP9
Raw data of blots for Figure 6D (upper panel in MDA-MB-231 cells)
